# Habitual tea consumption and 5-year incident metabolic syndrome among older adults: a community-based cohort study

**DOI:** 10.1186/s12877-021-02707-8

**Published:** 2021-12-19

**Authors:** Xing-Xuan Dong, Rui-Rui Wang, Jie-Yu Liu, Qing-Hua Ma, Chen-Wei Pan

**Affiliations:** 1 School of Public Health, Medical College of Soochow University, 199 Ren Ai Road, Suzhou, 215123 China; 2 The 3rd People’s Hospital of Xiangcheng District, Suzhou, China

**Keywords:** Metabolic syndrome, Tea consumption, Older adults, Cohort study

## Abstract

**Background:**

The effect of tea consumption on metabolic syndrome (MetS) remains controversial. The objective of this study is to examine the prospective association of tea consumption with 5-year incident MetS among aged population in China.

**Methods:**

This analysis included 3005 Chinese adults aged 60 years or older who were free of MetS at baseline examination. MetS was defined according to the National Cholesterol Education Program-Adult Treatment Panel III. Information regarding tea consumption was collected via an interviewer-administrated questionnaire. The prospective associations between tea consumption at baseline and 5-year incident MetS, as well as its individual components, were assessed by multiple logistic regression models.

**Results:**

Of the 3005 participants free of MetS at baseline, 406 participants (cumulative incidence: 13.5%) developed MetS at the 5-year follow-up examination. In multiple logistic regressions, 5-year cumulative incidence of MetS was found to be higher in those who drank tea more than 5 times per week as compared with non-habitual drinkers (OR = 1.38, 95% CI: 1.05-1.82; *P* = 0.02). This relationship still existed in men (OR = 1.43, 95%CI: 1.00-2.01; *P* = 0.05) when stratified by gender. Among the five major components of MetS, low high-density lipoprotein cholesterol was observed in men, while high body mass index, elevated blood pressure and the presence of diabetes mellitus were significant in women.

**Conclusions:**

High-frequent tea consumption increased the risk of MetS among older Chinese adults. These findings may add novel knowledge to the current studies regarding the controversial effect of tea consumption on cardiovascular and metabolic health among the aged population.

## Background

Metabolic syndrome (MetS) is a global health concern associated with cardiovascular diseases, common cancers, increased mortality and great socieconomic burden, particularly in aged populations. The prevalence of MetS has increased over recent decades, reaching alarming rates worldwide [1, 2]. In 2016, the World Health Organization estimated that one-fifth of the world’s adult population will develop MetS during their lifespan and the impact on their wellbeing and the health systems is tremendous. Thus, understanding its modifiable risk factors is crucial for formulating population-based intervention strategies for this condition.

Tea, the dried leaves of the plant *Camellia sinensis*, is a popular beverage worldwide. Recent evidence has suggested that tea consumption was associated with the risk of MetS but the effect seemed to be controversial. While some studies concluded that tea consumption is protective for MetS [3, 4], others reported non-significant associations [5, 6]. Furthermore, one study found that frequent consumption of tea increased the risk of MetS in rural China [7].

China is the most populous country all over the world and may have the greatest burden of MetS [8]. It is estimated that one-third of the Chinese adults are affected by MetS [9]. Meanwhile, tea, especially green tea, is very popular among middle-aged to elderly Chinese adults. Suzhou is one of the most important green tea producing areas in China, where tea drinking is a common lifestyle habit among general populations. In addition, the existing conflicting associations of tea and MetS are likely due to the lack of longitudinal study design. The purpose of this study is to explore the prospective associations between tea consumption and 5-year incidence of MetS in a community-based cohort study on older adults in Suzhou of China. The findings might be important for formulating non-pharmacological strategies for supplementing the management of MetS and its associated complications, especially in areas where tea drinking is a widely accepted cultural practice.

## Materials and methods

### Study population

The Weitang Geriatric Diseases study is a community-based study conducted in Weitang town among older adults aged 60 years or older in Suzhou, China. Detailed information about the study has been published elsewhere [10–13]. In brief, participants in the Weitang town of Suzhou were invited via invitation letters and were screened according to local official records. After excluding migrated residents and participants who had been living there less than 6 months, we enrolled 5613 adults who were considered to be eligible to participate. In 2014, 4611 eligible adults attended the baseline clinical examinations, among whom 4579 had complete data on interviewer-administered questionnaires including an Abbreviated Mental Test (AMT), underwent anthropometric examinations, and blood sample analyses. Five years later, these 4579 participants with complete data in the baseline study were invited to participate in the follow-up examination. If necessary, our study team conducted home visits or revisits to participants who were absent at the follow-up examinations to encourage participation. Those who moved away and did not provide updated contact information, refused to participate, or died before commencement of the 5-year follow-up examination were excluded from the follow-up study. Death of previous participants was confirmed through official death registration forms.

For the recruited participants, informed consent forms in writing duly were collected before their examination. The baseline and follow-up examinations of the Weitang Geriatric Diseases study followed the tenets of the Helsinki Declaration and were approved by the Institutional Review Board of Soochow University.

### Clinical examinations

The measurements of weight, height and blood pressure (BP) were performed using standardized methods. Body weight was measured to the nearest 0.1 kg without shoes. Height was measured in the standing position without shoes. Body mass index (BMI) was calculated as weight in kilograms divided by the square of height in meters (kg/m^2^). BP was measured 3 times or more after at least 5 min intervals of rest by an automatic blood pressure monitor (Dinamap model Pro Series DP110X-RW, 100 V2; GE Medical Systems Information Technologies, Inc., Milwaukee, Wisconsin, United States) and the value of BP was calculated from the average of the last two readings. The levels of high-density lipoprotein cholesterol (HDL-C), triglycerides (TG) and fasting plasma glucose (FPG) were determined from frozen blood samples collected and analyzed with the standard laboratory assays by laboratory technicians.

### Definition of MetS

We followed the National Cholesterol Education Program-Adult Treatment Panel III to define MetS based on the following five conditions [14]: (a) BMI of 25 kg/m^2^ or more; (b) BP of 130/85 mmHg or higher or on anti-hypertension medications; (c) blood TG ≥ 150 mg/dL (1.7 mmol/L); (d) blood HDL-C of lower than 40 mg/dL in men and 50 mg/dL in women; (e) fasting plasma glucose ≥7.0 mmol/L or with a history of diabetes mellitus [15]. If an individual was affected by three out of the above five conditions, he or she was considered to have MetS [16].

### Measurement of tea consumption

Detailed information on tea consumption of the participants was collected at recruitment stage by a trained research assistant through the survey. Participants responded to the question “Do you usually drink tea?” Those responded “no” were identified as non-habitual tea drinkers. Habitual tea drinkers are defined by tea consumption of 120 mL/d or more for at least 1 year. The frequency of tea drinking over the past 12 months was classified into “1-5 times/week” and “> 5 times/week”, tea type was grouped into green tea and other tea since most of the habitual tea drinkers drank green tea in Suzhou, China. The duration of tea drinking was categorized into “1-15 years”, “16-30 years” and “> 30 years”. Detailed information regarding the cohort’s tea drinking behaviors at baseline has been published elsewhere [17, 18].

### Covariates

Participants’ baseline information including socio-demographic characteristics (age, gender, education level, marital status, working status and monthly income), lifestyle-related habits (current smoking, alcohol consumption and physical activity) was collected in the baseline examinations. Marital status was defined as living “with” spouse or “without”, education level was determined into “primary education or below” or “secondary education or above”, and monthly income was made into three groups: “1000 Chinese Yuan (CNY) or less”, “1001-3000 CNY” and “more than 3000 CNY”.

### Statistical analysis

Continuous and categorical variables were presented as mean ± standard deviation (SD) and frequency (percentage), respectively. Characteristics of participants who were habitual tea drinkers versus non-habitual tea drinkers were compared using Student *t*-test or Chi-square test for continuous and categorical variables, respectively. Linear trends of individual MetS components across the frequency of tea consumption (0 times/week, 1-5 times/week, > 5 times/week) were investigated by Chi-square trend test. Two multiple logistic regression models were established to assess the relationship between tea consumption at baseline and the 5-year cumulative incidence of MetS as well as its individual components including high BP, high BMI, diabetes mellitus, low HDL-C and high TG, adjusting for potential confounders. Sensitivity analysis was used to select the most influential confounding factors. Model 1 adjusted for age and gender. Model 2 additionally controlled for initial BMI, educational level, monthly income and marriage status. Effect estimates including odds ratios (ORs) and their corresponding 95% confidence intervals (CIs) were presented. Gender-stratified analysis was also performed based on the same models. Interaction effects (different combinations of tea consumption and age, gender, education, socioeconomic status and other lifestyle habits such as smoking status, alcohol intake and physical activity) were investigated and excluded if the effects were not statistical significant. A two-sided *P*-value of less than 0.05 was considered statistically significant. Statistical analyses were performed using the SPSS version 21.0 (SPSS Inc., Chicago, IL, USA).

## Results

Among the 4579 participants with complete data in the baseline survey, we excluded 183 (4.0%) participants who had died prior to the 5-year follow-up examination and 526 (11.5%) participants who were lost to follow-up or deceased. The mean age of the participants at follow-up examinations was 70.7 ± 5.7 years (range: 65.0-76.4 years). Among the 3870 participants who successfully attended the follow-up examinations, 779 with MetS at baseline and 86 who had missing information for MetS features in the follow-up period were excluded as well, leaving 3005 participants available for the current prospective analyses ultimately. In the remained 3005 participants free of MetS at baseline survey, there were 1077 habitual and 1928 non-habitual tea drinkers, respectively. Table 1 compares the baseline characteristics between habitual and non-habitual tea drinkers. In general, habitual tea drinkers were more likely to be men (*P* < 0.001), current smokers (*P* < 0.001), alcohol drinkers (*P* < 0.001), and better educated *P* < 0.001). They also tended to live with spouse (*P* < 0.001), have higher income (*P* < 0.001), and have more physical activity (*P* < 0.001).

**Table 1:** Characteristics of study participants according to tea consumption habits

Characteristic	All persons (***n*** = 3005)	Non-habitual tea drinkers (***n*** = 1928)	Habitual tea drinkers (***n*** = 1077)	***P*** value
**Baseline characteristics**				
Gender (women), n (%)	1458(48.5)	1297(67.3)	161(14.9)	< 0.001
Age, mean(SD), years	67.3(5.7)	67.5(5.9)	67.0(5.4)	0.01
Living with spouse, n (%)	2506(83.4)	1567(81.3)	939(87.2)	< 0.001
Primary and below education, n (%)	2597(86.4)	1766(91.6)	831(77.2)	< 0.001
Monthly income, n (%)				< 0.001
≤ 1000 CNY	1682(56.0)	1177(61.0)	505(46.9)	
1001–3000 CNY	1101(36.6)	656(34.0)	445(41.3)	
> 3000 CNY	222(7.4)	95(4.9)	127(11.8)	
Current smoking, n (%)	844(28.1)	271(14.1)	573(53.2)	< 0.001
Alcohol consumption, n (%)	749(24.9)	252(13.1)	497(46.1)	< 0.001
Physical activity, n (%)	1273(42.4)	754(39.1)	519(48.2)	< 0.001
Working, n (%)	1135(37.8)	671(34.8)	464(43.1)	< 0.001
SBP, mean(SD), mmHg	142.4(19.6)	143.9(19.6)	139.6(19.3)	< 0.001
DBP, mean(SD), mmHg	84.9(11.3)	84.8(11.3)	85.1(11.3)	0.46
BMI, mean(SD), kg/m^2^	22.9(4.9)	22.8(5.8)	23.1(2.4)	0.11
HDL-C, mean(SD), mmol/L	1.6(0.4)	1.6(0.4)	1.5(0.4)	< 0.001
TG, mean(SD), mmol/L	1.1(0.5)	1.1 (0.5)	1.1(0.6)	0.44
FPG, mean(SD), mmol/L	5.4(0.9)	5.4(0.9)	5.4(0.9)	0.24
History of hypertension, n (%)	1481(49.3)	932(48.3)	549(51.0)	0.17
History of diabetes mellitus, n (%)	120(4.0)	76(3.9)	44(4.1)	0.85
**Follow-up characteristics**				
SBP, mean(SD), mmHg	148.9(21.0)	150.1(21.2)	146.6(20.4)	< 0.001
DBP, mean(SD), mmHg	82.1(11.5)	81.6(11.5)	83.1(11.3)	0.001
BMI, mean(SD), kg/m^2^	23.6(3.6)	23.5(3.8)	23.8(3.0)	0.05
HDL-C, mean(SD), mmol/L	1.6(0.4)	1.6(0.4)	1.5(0.4)	< 0.001
TG, mean(SD), mmol/L	1.2(0.7)	1.3 (0.7)	1.2(0.7)	0.27
FPG, mean(SD), mmol/L	5.7(1.0)	5.6(0.9)	5.7(1.1)	0.17
Metabolic syndrome, n (%)	406(13.5)	263(13.6)	143(13.3)	0.78

*SD* Standard deviation, *CNY* Chinese Yuan, *SBP* Systolic blood pressure, *DBP* Diastolic blood pressure, *BMI* Body mass index, *HDL-C* High-density lipoprotein cholesterol, *TG* Triglycerides, *FPG* Fasting plasma glucose

Among the 3005 participants free of MetS at baseline, 406 participants (cumulative incidence: 13.5%) developed MetS at the 5-year follow-up examination. We found that increased frequency of tea consumption was associated with decreased systolic BPs and decreased HDL-C levels (all *P* for trend < 0.001). Other components such as BMI, diastolic BPs, blood FPG and TG were not significantly related to the frequency of tea consumption.

**Fig. 1:**
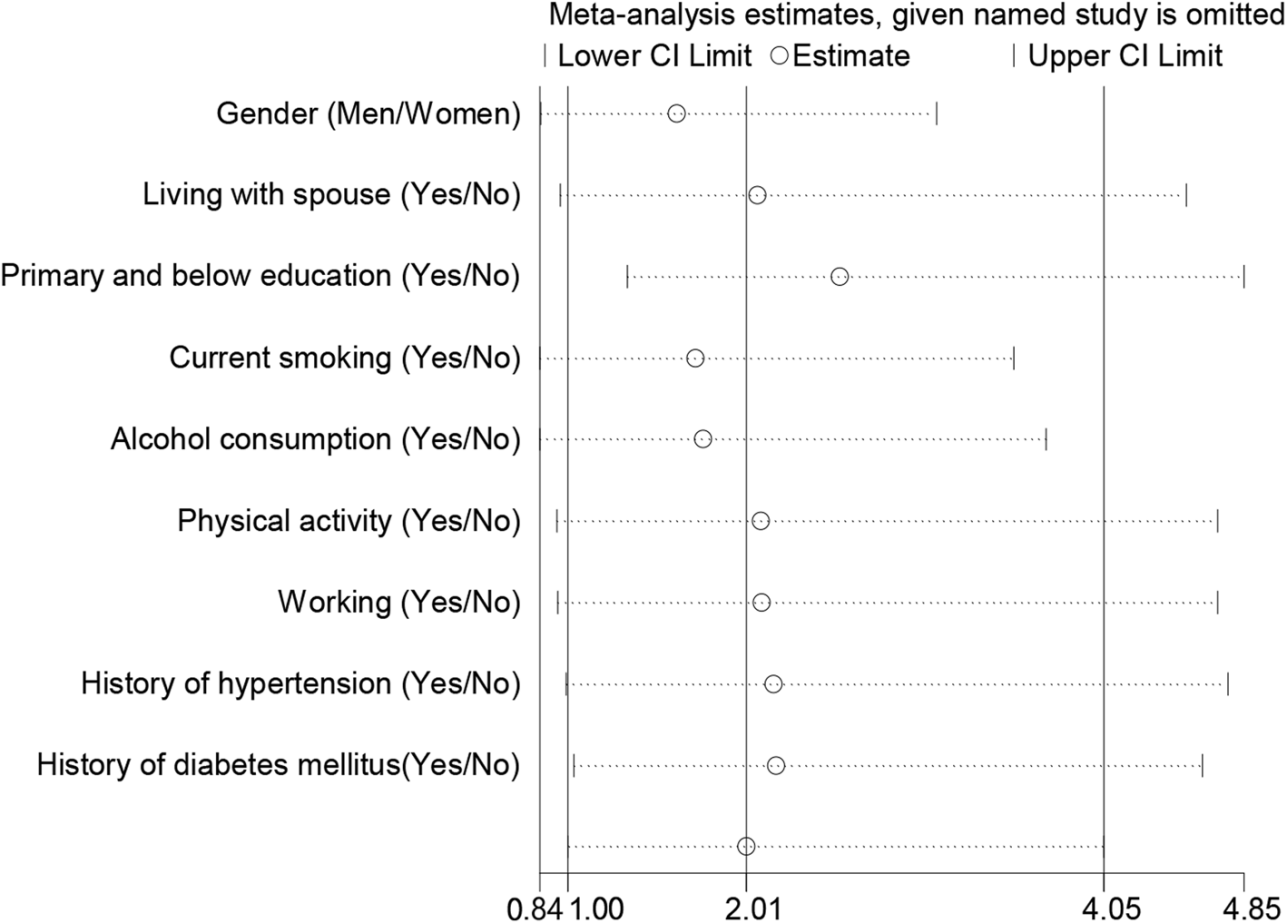
Sensitivity analysis of different characteristics and tea consumption habits of the study participants

The results of sensitivity analysis are presented in Fig. 1. We selected age, gender, initial BMI, educational level, monthly income and marriage status as confounding factor to be controlled. Prospective associations between tea consumption and the incident MetS were further examined and the results are shown in Table 2. After adjusting for baseline demographics such as initial BMI, educational level, monthly income and marriage status (Model 2), habitual drinkers had a 31% increased risk of developing MetS during the 5-year follow-up period compared with non-habitual drinkers (OR = 1.31, 95% CI: 1.01-1.71; *P* = 0.04). However, the significant association was not observed either in men (OR = 1.38, 95%CI: 0.98-1.95; *P* = 0.06) or women (OR = 1.17, 95%CI: 0.76-1.79; *P* = 0.48). Correspondingly, 5-year cumulative incidence of MetS was found to be higher in those who drank tea more than 5 times per week (OR = 1.38, 95% CI: 1.05-1.82; *P* = 0.02) as compared with non-habitual drinkers. This relationship still persisted in men (OR = 1.43, 95%CI: 1.00-2.01; *P* = 0.05) but was not significant in women (OR = 1.28, 95%CI: 0.79-2.08; *P* = 0.32) when stratified by gender. The association between green tea consumption and incident MetS was non-significant in all study participants (OR = 1.27, 95% CI: 0.97-1.67; *P* = 0.09), men (OR = 1.26, 95% CI: 0.91-1.76; *P* = 0.17) and women (OR = 1.22, 95% CI: 0.75-1.99; *P* = 0.42). Participants who drank tea for 16 to 30 years were more likely to develop MetS than non-habitual drinkers (OR = 1.53, 95%CI: 1.02-2.28; *P* = 0.04). The results were significant in neither men (OR = 1.39, 95%CI: 0.85-2.28; *P* = 0.19) nor women (OR = 1.79, 95%CI: 0.87-3.71; *P* = 0.11).

**Table 2:** Association of tea consumption and related variables with metabolic syndrome

Characteristics	Model 1						Model 2					
	All		Men		Women		All		Men		Women	
	OR(95% CI)	***P*** value	OR(95% CI)	***P*** value	OR(95% CI)	***P*** value	OR(95% CI)	***P*** value	OR(95% CI)	***P*** value	OR(95% CI)	***P*** value
**Tea**^a^												
Non-habitual drinker	1		1		1		1		1		1	
Habitual drinker	1.37(1.06, 1.78)	0.02	1.38(0.99,1.94)	0.06	1.35(0.90,2.03)	0.15	1.31(1.01, 1.71)	0.04	1.38(0.98,1.95)	0.06	1.17(0.76,1.79)	0.48
**Frequency**												
0 times/week	1		1		1		1		1		1	
1–5 times/week	1.06(0.60, 1.86)	0.84	0.95(0.42,2.18)	0.91	1.17(0.53,2.54)	0.70	0.99(0.56,1.76)	0.98	0.95(0.41,2.16)	0.90	0.90(0.40,2.04)	0.80
> 5 times/week	1.44(1.09, 1.89)	0.01	1.43(1.01,2.01)	0.04	1.42(0.89,2.27)	0.14	1.38(1.05,1.82)	0.02	1.43(1.00,2.01)	0.05	1.28(0.79,2.08)	0.32
**Tea type**												
Non-habitual drinker	1		1		1		1		1		1	
Green tea	1.32(1.01, 1.73)	0.04	1.28(0.92,1.78)	0.15	1.42(0.89,2.26)	0.14	1.27(0.97, 1.67)	0.09	1.26(0.91,1.76)	0.17	1.22(0.75,1.99)	0.42
Other	1.29(0.76, 2.20)	0.35	1.47(0.71,3.04)	0.30	1.13(0.52,2.46)	0.77	1.25(0.73, 2.15)	0.41	1.57(0.75,3.27)	0.23	1.00(0.45,2.22)	1.00
**Duration (years)**												
0(Non-habitual drinker)	1		1		1		1		1		1	
1–15	1.15(0.72, 1.85)	0.55	0.97(0.50,1.86)	0.92	1.41(0.71,2.80)	0.32	1.09 (0.68, 1.75)	0.72	0.97(0.50,1.86)	0.92	1.27(0.62,2.59)	0.51
16–30	1.62(1.09, 2.41)	0.02	1.38(0.84,2.26)	0.20	2.34(1.17,4.69)	0.01	1.53(1.02, 2.28)	0.04	1.39(0.85,2.28)	0.19	1.79(0.87,3.71)	0.11
> 30	1.34(0.98, 1.85)	0.07	1.35(0.93,1.95)	0.12	1.02(0.49,2.12)	0.96	1.31(0.95, 1.80)	0.11	1.33(0.92,1.94)	0.13	0.94(0.44,1.97)	0.86

Model 1, adjusted for age, gender

Model 2, adjusted for age, gender, initial BMI, educational level, monthly income, marriage status, smoking status, alcohol intake, physical activity

*BP* Blood pressure, *BMI* Body mass index, *HDL-C* High-density lipoprotein cholesterol, T*G* Triglycerides, *OR* Odds ratio, *CI* Confidence interval

^a^Tea consumption habits were collected at baseline

**Fig. 2:**
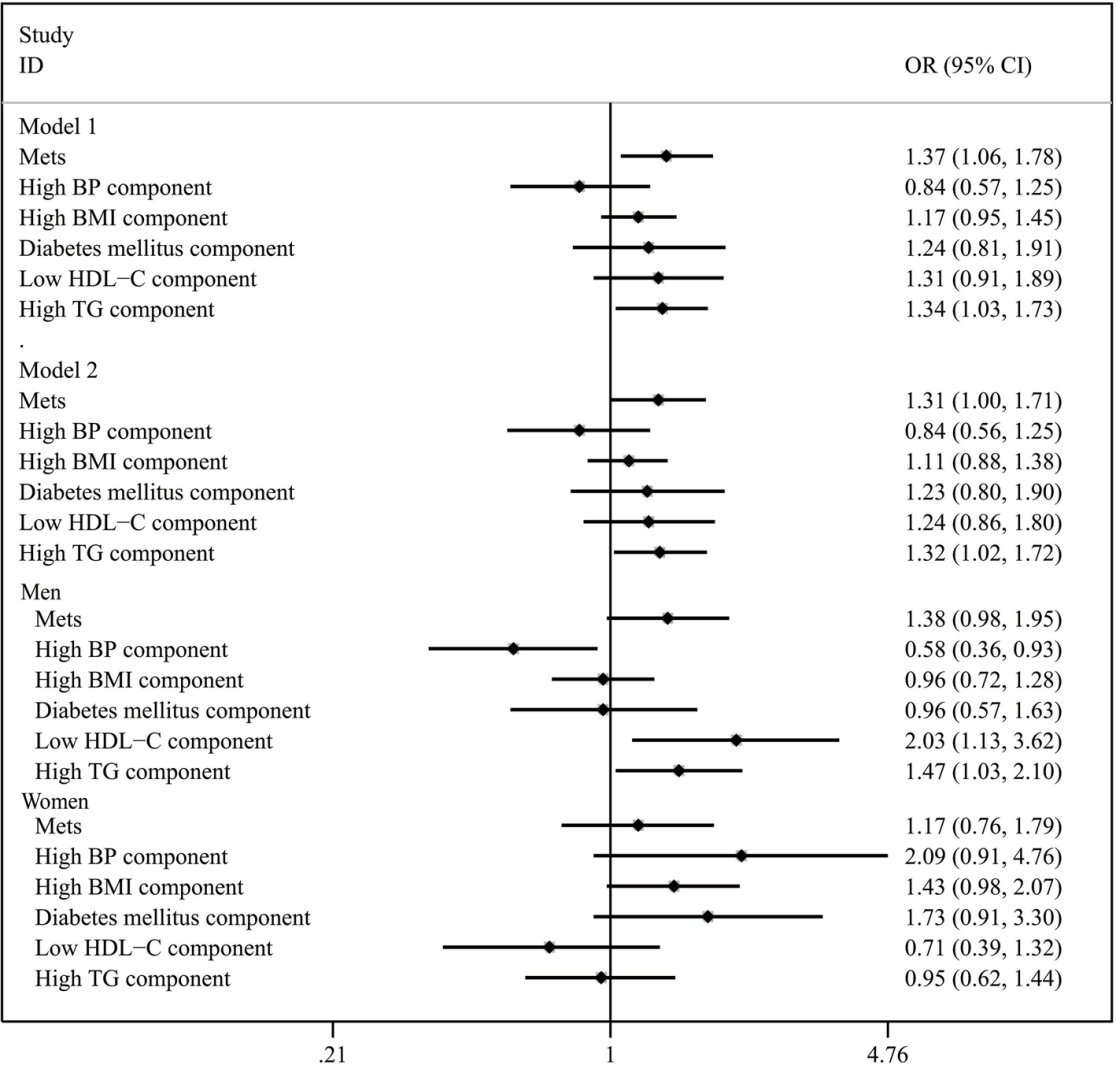
Risk of developing metabolic syndrome and its components according to tea consumption habits. BP, blood pressure; BMI, body mass index; HDL-C, high-density lipoprotein cholesterol; TG, triglycerides; OR, odds ratio; CI, confidence interval. *Tea consumption habits were collected at baseline. Model 1, adjusted for age, gender. Model 2, adjusted for age, gender, initial BMI, educational level, monthly income and marriage status

Figure 2 illustrates the prospective associations between tea consumption and individual components of MetS. No significant relationship was observed between the tea consumption at baseline and incident high BP, high BMI, diabetes mellitus or low HDL-C levels. The presence of tea consumption at baseline was related to high TG in both Model 1 (OR = 1.34, 95% CI: 1.03-1.73; *P* = 0.03) and Model 2 (OR = 1.32, 95% CI: 1.02-1.72; *P* = 0.04) among the participants, and the significant association still exist in men after stratified by gender (OR = 1.47, 95% CI: 1.03-2.10; *P* = 0.03).

Considering that tea consumption was much more frequent in men than women, we performed a gender-stratified analysis and the results are depicted in Fig. 1. Gender could be a possible influencing factor on the associations between habitual tea consumption and individual components of MetS. We found that low HDL-C levels were related to tea consumption in men (OR = 2.03, 95% CI: 1.11-3.69; *P* = 0.02). However, in women, tea consumption was positively associated with high BMI and elevated BP. Additionally, tea consumption was related with the presence of diabetes mellitus among women in Model 2 (OR = 2.02, 95% CI: 1.07-3.83; *P* = 0.03). The interaction effects between tea consumption and other variables were not observed.

## Discussion

In this 5-year prospective study on older Chinese adults, increased risk of developing MetS was observed in those who drank tea more than 5 times per week as compared with non-habitual drinkers. A dose-response relationship was also observed with increased frequency of green tea consumption being associated with a higher risk of MetS. In addition, the association of tea consumption and individual components of MetS varied between men and women. These findings added novel knowledge to the current literatures regarding the controversial effect of tea consumption on cardiovascular and metabolic health among the elderly.

The main finding of our study was different from most studies, which reported beneficial effects of tea consumption for MetS and some of its individual components [3, 4, 19]. A systematic review and meta-analysis summarized the findings of 6 observational studies and demonstrated that tea consumption were associated with a reduced risk of MetS [20]. Meanwhile, null associations were also reported in some other studies [5, 6, 21]. Weak relation of tea with MetS pointed towards the potential importance of composition of polyphenols and the types of tea consumed, and these observations needs to be confirmed in well-designed cohort studies. Based on the results of our analysis, there is, however, an increased risk of developing MetS in older adults who drank tea more frequently. Particularly, we found that habitual drinkers who drank tea more than 5 times per week were more susceptible to MetS. In addition, the relationship remained significantly different among men. In our study, women were less likely to be habitual tea drinkers compared with men, which reduced the sample size of women and may mask the association between tea consumption and MetS. Moreover, gender-stratified analysis may weaken the statistical power as the sample size was reduced by nearly half, making the effect of tea consumption on MetS insignificant either in men or women. Previous results indicated the deleterious effects of tea consumption, which was independently associated with an increased risk of kidney stones in Northern Chinese [22]. Concentrated herbal extracts from green tea may increase the burden of liver metabolism and may not be free of adverse effects under certain circumstances [23]. Additionally, about 80-85% of oxalate in diets of Chinese individuals was derived from tea and coffee [24], as a result, heavy tea-drinking habits will increase the excretion of urinary oxalic acid, which may lead to kidney stone formation, and eventually increase the burden of circulation. Similar results were not found when stratified by tea drinking duration. We considered that the information which relied on the subjective judgment and recall of the participants may lead to misclassification and information bias, thus may have distorted the effect estimates of tea consumption on MetS. In addition, perennial tea drinking habits may change due to some influential factors, such as health education and disease. What’s more, the information about the preparation method of tea (brewing or boiling) and the container of tea drinking was not available, so the serving size of tea may not be described accurately. Moreover, we found that habitual drinkers were more likely to be alcohol consumers, as shown in Table 1. The baseline characteristics of the 709 participants who did not enter the analysis were not different from those who entered the analysis. In rural China, having tea after taking alcohol is thought to be able treat effects of drunkenness or hangover. By leading the toxic acetaldehyde converted from alcohol to the kidney along with tea absorption before it decomposes, tea consumption after drinking could damage the kidney and finally disrupt normal blood flow [25]. Further studies are warranted to validate these hypotheses.

With regards to the association between tea consumption and individual components of MetS, we observed a gender difference. Low HDL-C levels were related to tea consumption in men while tea consumption was positively associated with high BMI, elevated BP and the presence of diabetes mellitus in women. The mechanisms underlying this phenomenon might be attributed to different lifestyles between men and women. For instance, women are significantly less physically active than men and obese women are most functionally impaired, and had lower perceived behavioral control toward physical activity [26]. Apart from this, one survey also found that men were more likely to smoke and drink alcohol [27]. Physical activity, smoking and alcohol intake are well-established risk factors for MetS and thus may modify the tea-MetS association between men and women.

The public health implication of our findings needs to be discussed. Tea consumption is traditionally considered to be a promising non-pharmacological strategy for supplementing the management of hypertension, obesity or diabetes, especially in places where tea drinking is a widely accepted cultural practice. In the era of rapid population aging and high prevalence of chronic diseases, tea drinking is regarded as an inexpensive and applicable dietary practice, which may be clinically relevant and show its public health importance. However, our study indicated that these beneficial effects need carefully re-consideration, especially when the drinkers are restricted to older adults. Some other aspects such as frequency of drinking, way of preparations and time for drinking should also be taken into consideration. The findings might be important for formulating non-pharmacological strategies for supplementing the management of MetS.

Our study is a community-based cohort study with a large sample size and a reasonable follow-up rate. Therefore, we provided a more cogent evidence than case-control or cross-sectional studies. Several limitations should also be acknowledged. First, the included participants were restricted to older Chinese adults who lived in the eastern part of China with proportionately high prevalence and incidence of MetS and extensive extrapolation of the findings to other populations needs further clarification. Second, tea consumption was self-reported and thus may result in recall biases, especially in older adults with cognitive decline. Finally, although we had controlled for a wide range of confounders, the possibility of unmeasured and residual confounding such as chronic diseases or medication treatment, the type of diet and dietary patterns, and whether malnutrition was involved that might have contributed to the risk of MetS, which might distort the associations observed in this study. Diet in Jiangnan region is healthier than that in other regions. On the one hand, residents are used to cooking with squeezed rapeseed oils, which is rich in Omega-6 and contributes to reducing total mortality [28–30]. On the other hand, due to the sufficient rainfall and fertile plains, Jiangnan residents often eat abundant aquatic products and fresh vegetables and fruits [31]. More studies are required to confirm the findings from this current study.

## Conclusions

In conclusion, increased risk of developing MetS was observed in high-frequent tea drinkers among older adults. Further well-designed cohort studies with more precise measurement of tea consumption and long-term follow-up duration are warranted to replicate our findings and the mechanisms underlying the adverse effect of tea consumption needs to be elucidated.

## Data Availability

The datasets used and/or analyzed during the current study are available from the corresponding author on reasonable request.

## Abbreviations

MetS: Metabolic syndrome
BMI: Body mass index
HDL-C: High-density lipoprotein cholesterol
TG: Triglycerides
FPG: Fasting plasma glucose
